# Human Anatomy

**Published:** 1849-09

**Authors:** 


Art. V.—*Human Anatomy.* By Jones Quain, M. D. Edited by Richard Quain, F. R. S., and William Sharpey, M. D., F. R. S., Professors of Anatomy and Physiology in University College, London. First American from the fifth London edition. Edited by Joseph Leidy, M. D. 2 vol. 8vo. Philadelphia: Lea & Blanchard. 1849.

A press of matter has prevented an earlier notice of this long expected and truly valuable edition of *Quain's Anatomy*—a work of too high standing in the estimation of the profession, to be profited by our poor praise.

The original treatise has been materially altered.

"The whole of the Section on General Anatomy is rewritten.

"The Descriptive Anatomy of the Osseous System has undergone various alterations, and some portions, including those which treat of the Formation and Growth of the several Bones of the Skeleton, belong exclusively to this edition. The description of the Articulations has been subjected to a complete revisal.

"Under the head of the Muscular System, many additions have been made; among which may be especially mentioned the account of the variations of form and attachment observed in individual muscles. Several parts have been re-written; but the paragraphs headed 'Dissection' and 'Action of Muscles' are printed from the preceding edition with scarcely any alteration.

"The principal changes to which the section on the Vascular System has been subjected, occur in the description of the Arteries. The history of each of the larger arteries has been recast, and a statement of the varied forms which these vessels present in different cases has been abridged from a special treatise published by one of the Editors.

"In the remainder of the work, including the description of the Brain, Nerves, and Organs of the Senses, the Heart, with the Digestive, Respiratory, Urinary, and Generative Organs, little or nothing remains of the former editions.

"The Surgical Anatomy, which has been introduced partly in connexion with the history of the principal arteries and partly at the end of the work, has likewise been written for the present edition."

Dr. Sharpey has contributed those portions of the work embraced under the heads of *General Anatomy;* and *Descriptive Anatomy* of the Brain, Heart, Organs of Respiration, Voice and Digestion, and the Genito-Urinary Apparatus: while to Dr. Quain has been assigned "the remaining portion of the *Descriptive Anatomy*, comprehending the Bones, Muscles, Articulations, Fasciæ, Vessels, Nerves and Organs of the Senses, as well as the *Surgical Anatomy* of the different regions."

A part of each of the Editors' labors was performed by their Junior Colleagues in the Anatomical Department of the University College, viz: Mr. Ellis, Mr. Potter and Mr. Marshall. So that, in fact, it is the united production of *five* accomplished Anatomists. There is not, however, to be found in its pages any evidences of such a multiplicity of editors ; on the contrary, the unity and completeness of every department, is almost a matter of marvel.

We cannot too highly commend the great research displayed by Dr. Sharpey, in his contributions to the subject of *General Anatomy*—particularly that portion of it relating to the structure and development of Bone, and the ultimate structure of Muscular Fibre.

We cannot refrain from giving a few extracts on these and other topics.

*Formation and growth of bone.*—The foundation of the skeleton is laid at a very early period, for among the parts that appear soonest in the embryo, we distinguish the rudiments of the vertebræ and base of the skull, which afterwards form the great median column to which the other parts of the bony fabric are appended. But it is by their outward form and situation only, that the parts representing the future bones are then to be recognized ; for at that early period they do not differ materially in substance from the other structures of the embryo, being like these, made up of granular corpuscles or elementary cells united together by a soft amorphous matter or blastema. Very soon, however, they become cartilaginous, and ossification in due time beginning in the cartilage and continuing to spread from one or from several points, the bone is at length completed.

But while it is true with respect to the bones generally that their ossification commences in cartilage, it is not so in every instance. The tabular bones, forming the roof of the skull, may be adduced as a decided example to the contrary ; in these the ossification goes on in a membranous tissue quite different in its nature from cartilage ; and even in the long bones, in which ossification undoubtedly commences, and to a certain extent proceeds in cartilage, it will be afterwards shown that there is much less of the increment of the bone really owing to that mode of ossification than is generally believed. It is necessary, therefore, to distinguish two species or modes of ossification, which for the sake of brevity may be called the *intramembranous* and the *intracartilaginous.*

*Ossification in membrane.*—The tabular bones of the cranium, as already stated, afford an example of this mode of ossification. The base of the skull in the embryo is cartilaginous; but in the roof, that is to say, the part comprehending the parietal, the upper and greater part of the frontal, and a certain portion of the occipital bones, we find (except where there happen to be commencing muscular fibres) only the integuments, the dura mater, and an intermediate membranous layer, which differs from cartilage in its intimate structure as well as in its more obvious characters, and in which the ossification proceeds.

The commencing ossification of the parietal bone, which may be selected as an example, appears to the naked eye in the form of a net work, the little bars or spicula of bone running in various directions, and meeting each other at short distances. By and by the ossified part, becoming extended, gets thicker and closer in texture, especially towards the centre, and the larger bony spicula which now appear, run out in radiating lines to the circumference; the ossification continuing thus to spread and consolidate until the parietal meets the neighboring bones, with which it is at length united by suture.

The adjoining figure (23,) represents the parietal bone of an embryo sheep, about two inches and a half long, and shows the character of the ossification as it appears when the object is magnified about twelve diameter. The bone is formed in membrane as in the human fetus, but a thin plate of cartilage rises up on its inside from the base of the skull. The ossification, however, is decidedly unconnected with the cartilage, and goes on in a membrane lying outside of it. The cartilaginous plate is not represented in the figure, but a dotted line, near the top, marks the height to which it reached, and from this it will be seen that the ossification extended beyond the cartilage. In the region of the frontal bone the cartilage does not even rise so high. In both cases its limit is well defined, and under the microscope it presents a decided contrast to the adjacent membrane.

When further examined with a higher magnifying power, the tissue or membrane in which the ossification is proceeding, appears to be made up of fibres and granular corpuscles, with a soft, amorphous or faintly granular uniting matter. The fibres have the characters of the white fibres, or rather fasciculi, of the cellular and the fibrous tissue, and are similarly affected by acetic acid. The corpuscles are for the most part true cells, with an envelope and granular contents; some about the size of blood-particles, but many of them two or three times larger. In certain parts the fibres, but in most the corpuscles, predominate; and, on the whole, the structure might be said to be not unlike that of a fibrous membrane in an early stage of development. The bone, seen by transmitted light, is dark and opaque, and near the growing edge it is decidedly granular.

On now observing more closely the bony processes or spicula at the circumference, where they shoot into the membrane (as in fig. 24), it will be seen, as you trace them into the soft tissue, that they gradually lose their opaque and granular character, indicative of earthy impregnation, and are prolonged a little way into the membrane, in the form of bundles of transparent fibres, having all the characters of those of fibrous tissue. These fibres are in some parts closely gathered into thick bundles, but more generally the fasciculi are smaller, and irregularly interlaced or reticulated, with corpuscles lying between them; and we may often observe that where the earthy deposit is advancing to invade the fibres, the recently and as yet imperfectly calcified bone with which they are continuous, presents a similar open and coarsely reticular structure; though the older, harder, and more opaque part is comparatively solid and compact. The appearance referred to is especially well seen at those places where a cross bridge of bone is being formed between two long spicula; we may there distinguish the clear soft fibres which have already stretched across the interval, and the dark granular opacity indicating the earthy deposit may be perceived advancing into them and shading off gradually into their pellucid substance without a precise limit.

It thus appears that in the intramembranous ossification the growing bone shoots into the soft tissue, in the form of transparent fibres, resembling those of fibrous texture, more or less intermixed with granular corpuscles, and that these fibres become charged with earthy salts. As to the cells or corpuscles, they certainly seem to be in some way involved in the ossification along with the fibres, but I am not able to say what precise share they have in the process. It has been supposed that they eventually form the lacunæ of the bone; but we shall enter upon this question afterwards.

As the bone extends in circumference it also increases in thickness, the vacuities between the bony spicula become narrowed or disappear, and at a more advanced period the tabular bones of the cranium are tolerably compact towards the centre, although their edges are still formed of slender radiating processes. At this time also numerous furrows are grooved on the surface of the bone in a similar radiating manner, and towards the centre these are continued into canals in the older and denser part, which run in the same direction. The canals, as well as the grooves, which become converted into canals, contain blood-vessels supported by processes of the investing membrane, which deposit concentric layers of bone within; and when thus surrounded with concentric laminæ, these tubular cavities are in fact the Haversian canals.

In regard to the ultimate structure of muscular fibre, Dr. Sharpey's observations are identical with those of Dr. Carpenter, published about the same time—both going to prove that the ultimate fibril consists of a series or string of cells, united end to end.

When a fibril thus completely insulated is highly magnified, it is seen to consist of a single row of minute particles, connected together like a string of beads. These particles (named "sarcous elements" by Bowman), when viewed with a magnifying power of 400 or 600, appear like little dark quadrangular and generally rectangular bodies, with bright intervals between them, as if they were connected together by some pellucid substance; but on closer examination, provided the defining power of the instrument is good, a very faint dark line or shadow will be discovered passing across the fibril in the middle of each of the bright spaces, and sometimes also a bright border may be perceived on either side of the fibril, so that each of the rectangular dark bodies appears then to be surrounded with a bright area having a similar quadrangular outline, and it may therefore be inferred that the pellucid substance encloses it on all sides. In short, it would seem that the elementary particles of which the fibril is made up, are little masses of pellucid substance, presenting a rectangular outline, and appearing dark in the centre. Their appearance, indeed, suggests the notion of minute vesicular bodies or cells, cohering in a linear series, the faint transverse marks between being the lines of junction. But although this idea very naturally presents itself, we must not assume that the reality of it is established. With a still higher magnifying power, the dark central part appears constricted in the middle, or looks as if it consisted of two portions joined together. When the focus is altered, the internal dark part becomes light; it is therefore evidently transparent, and its dark aspect is probably owing to its refracting the light differently from the surrounding substance. Minute pellucid objects, indeed, exhibit, when highly magnified, a dark centre surrounded by a bright halo, if viewed a little within the true focal distance; but the bright circumference of the muscular particles seems to be something more than can be accounted for in this way.

When the fibrillæ lie undisturbed in the fibre, the elementary particles of collateral fibrils are situated in the same transverse plane, and it is to this lateral coaptation of the particles that the transverse striping of the fibre is due. (See *b*, fig. 161.) Accordingly, the cross lines are not confined to the surface of the fibre, but may be seen throughout its entire thickness on successively deepening the focus of the microscope. The fibres moreover often show a tendency to cleave across in the direction of these lines, and even to break up into transverse plates or disks (fig. 162), which are formed by the lateral cohesion of the particles of adjacent fibrils. To make up such a disk, therefore, every fibril contributes a particle, which separates from those of its own fibril, but coheres with its neighbors on either side, and this with perfect regularity. Indeed, Mr. Bowman conceives that the subdivision of a fibre into fibrillæ is merely a phenomenon of the same kind, only of more common occurrence, the cleavage in the latter case taking place longitudinally in place of transversely: accordingly, he considers that the fibrillæ have no existance as such in the fibre, any more than the disks; but that both the one and the other owe their origin to the regular arrangement of the particles of the fibre longitudinally and transversely, whereby, on the application of violence, it cleaves in the one or in the other direction into regular segments.

It will be seen from the following extract, that the true arrangement of the *nerve-fibres* at their peripheral extremities, according to Dr. Sharpey, is not by any means what it is commonly supposed to be.

The results of modern microscopic discovery seemed for a time to lead to the conclusion that the fibres of nerves do not, strictly speaking, end in the tissues in which they are distributed, but merely dip into those tissues, as it were, and, after forming slings or loops of greater or less width, return sooner or later to the nervous trunks. The further progress of inquiry has, however, failed to establish the generality of this conclusion, and has even gone far to disprove the existence of the alleged mode of termination in various cases in which it had been previously held to take place; and, indeed, it must be admitted that the arrangement of the nervous fibres at their peripheral extremities is still but imperfectly understood.

In no case was the termination of nerves by loops more generally acknowledged than in voluntary muscle; but certain observations, very recently made known by Wagner, are calculated to throw considerable doubt on the opinion hitherto received, especially when viewed in connexion with the results of collateral inquiries respecting the nerves of various other textures. Wagner states that, in the muscles of the frog, the tubular nerve-fibres may be observed to be at last divided into fine branches, which appear (although he is not quite certain on this point) to perforate the sarcolemma of the muscular fibres, and to divide into still finer filaments, not more than 1-10,000th or 1-12,000th of an inch in size, that run between the muscular fibrillæ, where they elude further scrutity.

With regard to the plexiform arrangement of the terminal fibres of the nerves in the skin of the frog, our author speaks as follows:

Although the arrangement appears to the eye such as is described, further inquiries have shown that there is something beyond. Thus, fibres have been seen (by Todd and Bowman) passing from the plexus, through the superjacent layers of membrane towards the surface of the skin, and not visibly ending in loops, but becoming at last lost to view. In the eyelid of the frog, also, in which the plexiform arrangement of the fine nervous branches is readily seen, Henle observed nerve-fibres which ran singly a long way, and then disappeared, there being no evidence that they were continuous with others in the form of loops; some seemed to end abruptly, and this appearance, which Henle was disposed to consider fallacious, has since been described again by Hannover, who, moreover, saw other primitive fibres dividing into finer filaments, which were arranged into a plexus, and ultimately eluded the sight.

To these examples must be added the remarkable observations of Schwann on terminations of the nerves in the web or fin of the tadpole's tail. In that instance, as well as in the mesentery of amphibia, it appeared to Schwann that the ordinary primitive nerve-fibres, after separating from the fasciculi, divided into other fibres of much smaller size, and that the finer fibres resulting from this division, which were destitute of white substance, and wanted the dark outline, here and there presented little enlargements or nodules, from whence, again, delicate fibres spread out in various directions, and connect themselves in form of a network. Subsequent observation made by myself on the nerves of the tail of the tadpole, are confirmatory of those of Schwann. The fine fibres, which are derived from the division of the ordinary ones, want the bold, dark outline which usually marks the tubular fibres; they also present, here and there along their course, elongated corpuscles, like cell-nuclei, and, from their similarity in aspect to the gelatinous fibres, it might be supposed that they are really prolonged from gelatinous fibres mixed up with the tubular kind in the nervous branches; there can be no doubt, however, as to their source, for fine tubular fibres may be traced, which change their character as they proceed, lose their dark outline, and pass continuously into these pale nucleiferous fibres; moreover, many of the decidedly tubular fibres in this situation are marked with nucleus-like corpuscles. The tubular fibres might thus be represented as laying aside their white substance and dark outline before dividing or terminating, like those ending in the Pacinian bodies; but in the present case (of the growing tail of the tadpole) the pale fibres are, in reality, to be considered as an earlier condition of tubular fibres in progress of development. Schwann, who adopts this view, states that the pale fibres are the forerunners of tubular fibres, and he conceives that they are converted into the latter by acquiring the white substance (medullary sheath), and with this the dark outline. Still, whether perfect or not, these fine fibres must be capable of receiving and conducting sensorial impressions applied to the decidedly sentient membrane in which they are distributed.

The whole account of the nervous system throughout is clear and precise. We quote from this part of the work only the following additional paragraph, a good summary of our actual knowledge of the structure and function of the great sympathetic.

From what has been stated, it seems reasonable to conclude that nerve-fibres take their rise in the ganglia both of the cerebro-spinal and sympathetic nerves, and are, in both kinds of nerves, mixed with fibres of cerebral or spinal origin; that the ganglia are nervous centres which probably receive, through afferent fibres, impressions of which we are unconscious, and reflect these impressional stimuli upon efferent or motor fibres; that perhaps, even, certain motorial stimuli emanate from them, the movements excited by or through the ganglia being always involuntary, and affecting chiefly the muscular parts of the viscera, the sanguiferous, and perhaps the absorbent vessels; and that, in fine, the chief purpose served in the animal economy by the ganglia and ganglionic nerve-fibres, whether existing in acknowledged branches of the symppathetic, or contained in other nerves, is to govern the involuntary, and, for the most part, imperceptible movements of nutrition, in so far at least, as these movements are not dependent on the brain and spinal cord.

One more quotation, and we are done. It is in relation to a much controverted point, viz., the development of the capillary bloodvessels—which is now apparently in a fair way to be cleared up:

The smaller vessels and capillaries originate from nucleated cells similar to those which at first constitute the different parts of the embryo. The cell-wall or envelope of these cells shoots into the slender pointed processes, tending in different directions, so that they acquire an irregularly star-shaped or radiated figure. The prolongations from neighboring cells encounter one another, and join together by their ends; and the irregularly ramified or reticulated cavities thus produced, are the channels of the rudimental capillaries. In growing parts, where new vessels are formed in the vicinity of those already existing, not only do the processes of the stellate cells join those of neighboring cells, but some of them meet and join with similar pointed processes which shoot out from the sides of neighboring capillary vessels, and in this manner the new vessels are adopted into the existing system. The junctions of the cells with each other, or with capillary vessels, are at first of great tenuity, and contrast strongly with the central and wider parts of the cells; they appear then to be solid, but they afterwards become pervious and gradually widen, blood begins to pass through them, and the capillary network acquires a tolerably uniform calibre. The original vascular network may become closer by the formation of new vessels in its interstices; and this is effected by similarly-metamorphosed cells, arising in the areolæ, and joining at various points with the surrounding vessels, and also, according to Kölliker, simply by pointed off-shoots from the existing capillaries stretching across the intervals, and meeting from opposite sides, as when enlarged to form new connecting arches. From observations made on the fetal membranes of sheep, Mr. Paget has found that the mode of formation of capillaries described by Kölliker in batrachians, holds good also in mammiferous animals.

Of the main body of the work—*Descriptive Anatomy*—but little need be said. Much of it is the same in words with the former standard edition, and such as has been changed at all, has been changed for the better. On the whole we know of no work, which we would sooner see in the hands of every student of this branch of medical science than Sharpey & Quain's Anatomy.

